# The Establishment of Esophageal Precancerous Lesion Model by Using *p53* Conditional Knockout Mouse in Esophageal Epithelium

**DOI:** 10.1155/2020/4534289

**Published:** 2020-01-23

**Authors:** Lili Zhu, Yanyan Xu, Xinhuan Chen, Jiace Qin, Tingting Niu, Yanyan Zhu, Yanan Jiang, Simin Zhao, Kangdong Liu, Jing Lu, Ge Jin, Junfen Ma, Ziming Dong, Jimin Zhao

**Affiliations:** ^1^Department of Pathophysiology, School of Basic Medical Sciences, Zhengzhou University, Zhengzhou 450001, China; ^2^Henan Provincial Cooperative Innovation Center for Cancer Chemoprevention, Zhengzhou 450001, China; ^3^Department of Pathology, The Sixth People's Hospital of Zhengzhou, Zhengzhou 450000, China; ^4^Department of Dermatology, The Second Affiliated Hospital, School of Medicine, Xi'an Jiaotong University, Xi'an 710004, China; ^5^The China-US (Henan) Hormel Cancer Institute, Zhengzhou 450008, China; ^6^Department of Biochemistry and Molecular Biology, School of Basic Medical Sciences, Zhengzhou University, Zhengzhou 450001, China; ^7^Department of Clinical Laboratory, The First Affiliated Hospital of Zhengzhou University, Zhengzhou 450052, China

## Abstract

Understanding the molecular mechanisms of precancerous lesion of esophageal cancer is beneficial for early diagnosis and early treatment. The deletion of *p53* gene is common in esophageal cancer, but its pathogenesis is still unclear. An animal model is urgently needed to study the mechanisms of esophageal cancer and *p53* deficiency. KO mice (p53^flox/flox^.ED-L2-Cre^+/−^) and the corresponding control Loxp mice (p53^flox/flox^.ED-L2-Cre^−/−^) were obtained by crossing between the p53^flox/flox^ mice and ED-L2-Cre^+/−^ mice. Methylbenzylnitrosamine (NMBA) was injected subcutaneously to induce esophageal precancerous lesion of these two groups of mice. Hematoxylin and eosin staining analysis was performed to evaluate the number and extent of esophageal precancerous lesions in KO mice and Loxp mice at the 16th and 48th weeks. Immunohistochemistry analysis was used to detect the change of Ki67, P21, Bcl-2, and Bax proteins. The number and extent of esophageal precancerous lesions in KO mice were significantly increased compared with the control at the 16th and 48th weeks under the induction of NMBA. The Ki67, P21, Bcl-2, and Bax proteins also had cancer-related pathological characteristics. These results suggest that the esophageal precancerous lesion model was established under the combined effect of *p53* gene deletion in esophageal epithelium and NMBA, which could provide a new esophageal precancerous lesion model to explore the mechanism of precancerous lesions.

## 1. Introduction

Esophageal cancer is highly prevalent in developing countries [1] and it is classified into squamous cell carcinoma and esophageal adenocarcinoma according to histomorphological features, of which squamous cell carcinoma is the main type of diseases in China [2]. The pathogenesis of ESCC has not been studied so clearly that there are not effective prevention and treatment measures, which caused that ESCC has high morbidity and mortality [3]. The development of esophageal squamous cell carcinoma is a multistage, multistep progressive process involving simple hyperplasia and mild, moderate, and severe dysplasia and finally esophageal cancer [4]. At present, studies have shown that intervention in the precancerous lesions of esophageal cancer can effectively reverse the disease [5, 6]. Exploring the mechanism of precancerous lesions may be another way to solve the high mortality of esophageal cancer.

The occurrence and development of tumors are closely related to gene mutations. As known, p53 mutations occur in half of human cancers [7]. As a tumor suppressor, p53 can repair cells damage and clear cells that cannot be repaired [8]. Therefore, once p53 mutation or deletion occurs, which will lead to the loss of the control of cell proliferation and cell carcinogenesis. As one of the most common protein variants expressed in cancer cells [9], p53 mutations occur in more than half of colorectal cancers [10] and the loss of p53 is examined on gastric carcinogenesis [11]. There is also some evidence that p53 mutations or deletions are closely related to esophageal cancer. For example, aberrant p53 gene alleles are common genetic events in the pathogenesis of ESCC [12]; Krüppel-like factor 5 loss harboring mutant p53 leads to the formation of invasive tumors [13]. At present, most of the research on the role of p53 in esophageal cancer remains in the detection of in vitro experiments and clinical tumor specimens [14, 15], but the specific molecular mechanism has not been fully explored. Transgenic mice, such as p53-deficient mice, may provide advantages in revealing the pathogenesis of precancerous lesion of esophageal cancer.

The occurrence of esophageal cancer is not caused by a single factor, but it is the result of multiple genetic changes, multichannel regulation, and multifactor interactions [16]. Studies have confirmed that nitrite is prevalent in water, soil, and food in areas with high incidence of esophageal cancer [17–19]. Methylbenzylnitrosamine (NMBA) can specifically induce esophageal cancer in animals [20], which is currently recognized as a carcinogen for esophageal squamous cell carcinoma. In addition, there are also studies showing that using NMBA administration can induce squamous cell carcinoma in the mouse forestomach [21]. Therefore, in this study, the Cre/Loxp system was used to establish a model of p53-specific knockout mice (KO mice) in esophageal epithelial tissues, and then esophageal precancerous lesions were established in KO mice induced by NMBA. In addition, we also did a preliminary study on the mechanism of action of p53 in the development of esophageal precancerous lesions.

## 2. Materials and Methods

### 2.1. Cell Lines and Chemicals

The immortalized esophageal epithelial cells (SHEE cells) were generously supplied by Dr. Enmin Li (Institute of cancer Pathology, Shantou University Medical College). NMBA was obtained from East China University of Science and Technology (Shanghai, China) and the purity was 98% as determined by high-performance liquid chromatography.

### 2.2. KO Mice Model

**Figure 1:**
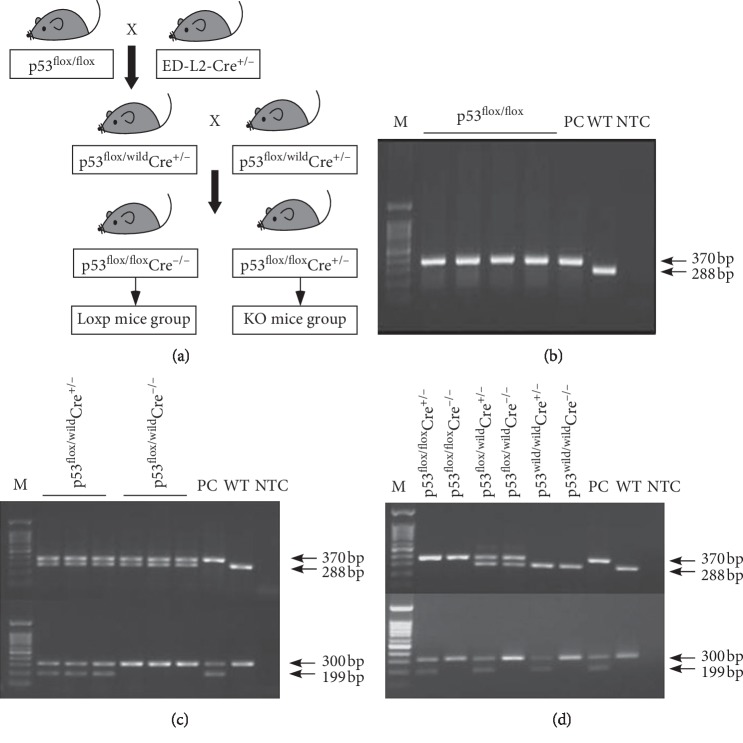
Breeding strategy and genotype identification. (a) Flow chart of breeding design for the mice with desired genotypes of p53^flox/flox^Cre^−/−^ and p53^flox/flox^Cre^+/−^ by using the mice with genotypes of B6.p53^flox/flox^ and ED-L2-Cre^+/−^. (b) The genotype of p53^flox/flox^ mice. (c) The genotype of F1 generation mice. (d) The genotype of F2 generation mice. M: marker; PC: positive control; WT: wild type control; NTC: no temple control.

The B6.p53^flox/flox^ mice (C57BL/6J) were introduced from the Jackson Laboratory of the United States. The ED-L2-Cre^+/−^ transgenic mice were gifts from the NCI (National Cancer Institute, USA) mouse library. The schematic of the genotypes used for crossing is shown in Figure 1(a). Briefly, B6.p53^flox/flox^ mice were crossbred with ED-L2-Cre^+/−^ mice to generate two genotypes mice: B6.p53^flox/wild^.ED-L2-Cre^+/−^ and B6.p53^flox/wild^.ED-L2-Cre^−/−^. The mice of genotype B6.p53^flox/wild^.ED-L2-Cre^+/−^ were mated with each other and screened out two genotypes of mice: B6.p53^flox/flox^.ED-L2-Cre^+/−^ (KO mice) and B6.p53^flox/flox^.ED-L2-Cre^−/−^(Loxp mice). These mice were housed under standard conditions (20 ± 2°C; 50 ± 10% relative humidity; 12 h light/dark cycles) and were provided with food and water ad libitum.

### 2.3. Identification of Mouse Genotypes by PCR

When mice were 3 weeks old, the tail biopsies were obtained and the genomic DNA were extracted using protease K. PCR genotyping was performed and identified by agarose gel electrophoresis. The primers used were as follows: p53 forward, 5′-CACAAAAACAGGTTAAACCCAG-3′ and reverse, 5′-AGCACATAGGAGGCAGAGAC-3′; L2-Cre enzyme forward, 5′-ACCAGCCAGCTATCAACTCG-3′ and reverse, 5′-TTACATTGGTCCAGCCACC-3′; reference gene forward, 5′-CTAGGCCACAGAATTGAAAGATCT-3′ and reverse, 5′-GTAGGTGGAAATTCTAGCATCATCC-3′. PCR conditions were as follows: predenaturation at 94°C for 3 min; 35 cycles at 94°C for 30 sec, 60°C for 30 sec, 72°C for 1 min; eventually extended at 72°C for 5 min; and kept cold at 4°C for holding standby.

### 2.4. Real-Time Fluorescence Quantitative PCR Assay

The esophagus of KO mice and Loxp mice was taken, and the mucosal layer was separated from the muscular layer and then homogenized separately. The total RNA was extracted by Trizol and reverse transcriptional reaction was performed. Then cDNA samples were prepared and used as a template for qPCR detection for p53 mRNA expression of mouse esophageal mucosa. The primers were as follows: p53, F-GTGAAGCCCTCCGAGTGTC and R-CAGGTGGAAGCCATAGTTGC; the housekeeping gene GAPDH(glyceraldehyde-3-phosphate dehydrogenase): F-AGGTCGGTGTGAACGGATTTG and R-TGTAGACCATGTAGTTGAGGTCA. PCR conditions were 30∼40 cycles at 95°C for 2 min, 95°C for 5 sec, and 60°C for 30 sec. The relative expression of the p53 mRNA level was analyzed using the average *C*_t_ value of every sample's gene by 2^−ΔΔCT^method.

### 2.5. Hematoxylin and Eosin Staining

The esophagus was taken and fixed with 10% formalin, embedded in paraffin, and cut into 4 *μ*m thick specimen sections. The slices were baked in a 65°C oven for 2 h. The sections were deparaffinized in xylene and rehydrated with graded alcohol. The slide was dipped into a box containing hematoxylin for 5 min and then it was rinsed in H_2_O for 3 min. The slide was differentiated by 1% hydrochloric alcohol and rinsed in H_2_O again and then stained with eosin solution for 10 min. After rinsing in water for 30 min, the sections were dehydrated with graded alcohol and cover slipped. All the sections were scanned using TissueFAXS (TissueGnostics GmbH, Vienna, Austria).

### 2.6. Evaluation of Histologic Grade

After being fixed in 10% neutral buffered formalin, all esophageal tissues were embedded in paraffin and cut into 4 *μ*m sections and then stained using hematoxylin and eosin (HE). According to the diagnostic criteria of epithelial dysplasia of the World Health Organization (WHO) in 2005, there are 5 histological categories: normal epithelium, simple hyperplasia, mild atypical dysplasia, moderate atypical dysplasia, and severe atypical dysplasia. Normal esophageal epithelium usually shows normal cell thickness and an orderly basal layer. Hyperplasia has a little thickening of the basal cell and keratin layers. Dysplasia is composed of increased cellular atypia, disorderly epidermal cells and basal layer cell, and more obvious thickening of the keratin layer. According to the degree of cytologic atypia, dysplasia was categorized as mild dysplasia (affect <1/3 of epithelium), moderate dysplasia (affect 1/3 to 2/3 of epithelium), and severe dysplasia (affect >2/3 of epithelium) [22]. The lesions at all levels from the esophagus of each mouse were counted and the total number of each histological category per group was recorded.

### 2.7. Immunohistochemistry

The expression of p53 protein in esophageal mucosa of KO mice and Loxp mice was detected, respectively, by immunohistochemical staining. Tissue sections are prepared in the same way as HE-stained sections. The slices were baked in a 65°C oven for 2 h. The sections were deparaffinized in xylene and rehydrated with graded alcohol. Then the microwave antigen repair technique was used with EDTA buffer (PH 9.0) or sodium citrate buffer (PH 6.0). Dripped in 3% hydrogen peroxide solution for 10 min, the sections were incubated with antibodies against p53 (1 : 100 dilution, Proteintech, USA), Ki67 (1 : 50 dilution, Fujian, China), P21 (1 : 50 dilution, Servicebio, China), Bax (1 : 100 dilution, Servicebio, China), and Bcl-2 (1 : 100 dilution, Servicebio, China) at 4°C overnight. After washing with TBST, the slides were incubated with HRP-conjugated secondary antibody at 37°C for 15 min. The peroxidase activity was detected with 2,4-diaminobenzidine. Then, the sections were counterstained with hematoxylin, dehydrated with graded alcohol, and cover slipped. All the sections were scanned by using TissueFAXS and analyzed by using HistoQuest 4.0 software. Hematoxylin staining was used as the reference index to confirm the cells. The intensity values of hematoxylin and DAB were calculated by software analysis. Then the boundary values of positive cells and negative cells were determined according to DAB intensity values. Finally, the percentage ratio of positive cells in each group was analyzed.

### 2.8. NMBA Carcinogenesis

The mice of genotype B6.p53^flox/flox^.ED-L2-Cre^+/−^ and B6.p53^flox/flox^.ED-L2-Cre^−/−^ were injected subcutaneously with NMBA (1 mg/kg) three times a week for 5 weeks and followed by observation. At the 16th weeks after the first injection, three mice in Loxp mice group and five mice in p53 KO mice group were euthanized. At the 48th week, the remaining thirteen mice of each group were sacrificed, and their esophagus was observed and embedded in paraffin.

### 2.9. RNA Interference

The siRNA targeting p53 was purchased from Shanghai GenePharma Co., Ltd. The cells were transfected with 8 *μ*l siRNA (20 *μ*M), 200 *μ*l jet prime buffer, and 8 *μ*l jet prime (Polyplus-transfection). After transfected for 24 h, the cells need to be observed and were harvested at 48 h after transfection. Subsequently, p53 expression was analyzed by Western blotting.

p53-homo-1# sense, 5′-GCAUCUUAUCCGAGUGGAATT-3′ and antisense, 5′-UUCCACUCGGAUAAGAUGCTT-3′; p53-homo-2# sense, 5′-GCUGUGGGUUGAUUCCACATT-3′ and antisense, 5′-UGUGGAAUCAACCCACAGCTT-3′; p53-homo-3# sense, 5′-CUACUUCCUGAAAACAACGTT-3′ and antisense, 5′-CGUUGUUUUCAGGAAGUATT-3′; Negative control sense, 5′-UUCUCCGAACGUGUCACGUTT-3′ and antisense, 5-ACGUGACACGUUCGGAGAATT-3′.

### 2.10. Western Blot Analysis

SHEE cells transfected by siRNA were collected through centrifugation and RIPA lysis buffer was added (50 nM Tris-base, 1%NP-40, 0.25%SOD, 150 mM NaCl, 1 mM EDTA, 0.1%SDS, 1 mM Na_3_VO_4_, 1 mM NaF, 1 mM PMSF, and 10 *μ*l protease inhibitor). Then cell protein lysates (50 *μ*g) were electrophoresed through 10% SDS-PAGE gel according to the protein concentration measured by a protein assay kit (Beyotime Institute of Biotechnology), and the protein bands were transferred to polyvinylidene difluoride (PVDF) membranes. The membranes were blocked with 5% skim milk for 1 h at room temperature. After being washed with TBS, the PVDF membranes were incubated with primary antibody against p53 (1 : 1000) at 4°C overnight. The next day, the membranes were incubated with HRP-IgG secondary antibody (1 : 10000) for 2 h at room temperature and were scanned by Odyssey Clx (Licor, US).

### 2.11. Anchorage-Independent Cell Growth Assay

The growth medium (Basal Medium Eagle, BME, Sigma-Aldrich, United Kingdom) supplemented with 10% FBS, 0.1% gentamicin, 1% glutathione, 9% sterile water, and 0.5% agarose was spread in a 6-well plate at a volume of 3 ml/well and stood still for 2 h. Meanwhile, p53-knockdown SHEE cells and control SHEE cells both treated by 25 *μ*g/ml NMBA for 48 h were resuspended in the growth medium (BME) supplemented with 10% FBS, 0.1% gentamicin, 1% glutathione, and 45% sterile water and then 0.3% agar was added in a top layer over a base layer. The cells were seeded at concentration 8000 cells/well of a 6-well plate and maintained at 37°C in a 5% CO_2_ incubator. Eight days later, the colonies were counted and photographed under a microscope by using the Image-Pro Plus software program (Media Cybernetics, Rockville, MD, USA).

### 2.12. Statistical Analysis

All quantitative data was expressed as mean ± standard deviation. Student's *t*-test and one-way analysis of variance by SPSS 17.0 (IBM Corp, Armonk, NY, USA) were used for statistical analysis. Statistics was considered to be significant when *p* < 0.05.

## 3. Results

### 3.1. Breeding Strategy and Genotype Identification

In order to obtain the target gene mouse, B6.p53^flox/flox^ mice were crossed to ED-L2-Cre^+/−^ mice and then the different gender offspring of the same genotype p53^flox/−^Cre^+/−^ (F1 generation mice) were mated with each other. Subsequently, the genotypes of p53^flox/flox^Cre^+/−^ mice (F2 generation mice) were obtained (Figure 1(a)). Meanwhile, the genotypes of mice were identified by PCR and agarose gel electrophoresis (Flox: 370 bp; Wild: 288 bp; Cre: 199 bp; Housekeeper gene: 300 bp) (Figures 1(b)–1(d)).

### 3.2. The Expression of p53 mRNA and Protein in the Esophageal Mucosa of p53 Knockout Mice Was Significantly Reduced

**Figure 2:**
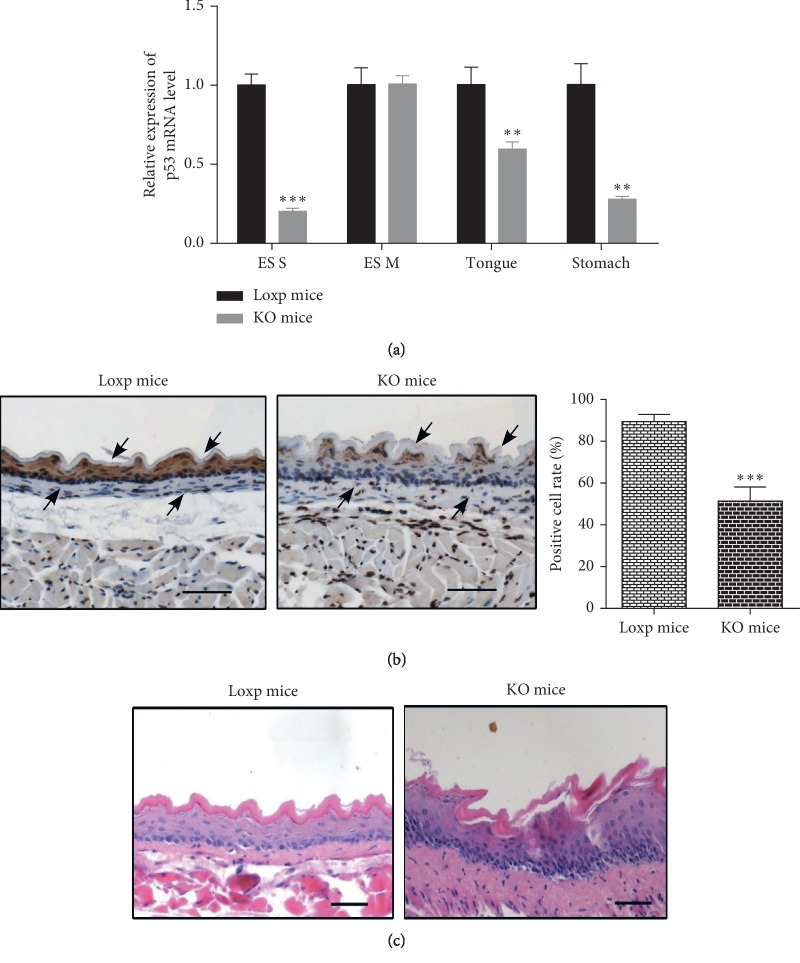
The expression of p53 in the esophageal mucosa of p53 KO mice was significantly reduced. (a) The p53 mRNA levels in esophageal mucosa, esophageal muscle layer, tongue, and stomach tissues of the two genotype mice (KO mice and Loxp mice) were detected by qPCR. Three mice were taken from each group, and each mouse had three replicates. ES S: esophageal mucosa; ES M: esophageal muscle layer (^*∗∗*^ means *p* < 0.01, ^*∗∗∗*^ means *p* < 0.001). (b) The expression of p53 protein was detected by immunohistochemistry analysis. P53 expressions stain a brown color and the nuclei are stained blue with hematoxylin. The staining of p53 was analyzed using HistoQuest 4.0 software. Arrows refer to the esophageal mucosa (*n* = 3, ^*∗∗∗*^ means *p* < 0.001). (c) HE staining of esophageal tissues in 20-week-old p53 KO mice and Loxp mice. Scale bar: 100 *μ*m.

To prove the knockout degree of *p53* gene, the total RNA was extracted from esophageal mucosa, esophageal muscular layer, tongue, and stomach tissues of 10-week-old KO and Loxp mice. The purity and concentration of RNA were determined and the reverse transcription reaction was performed. The reverse transcription reaction products were used as templates for QPCR. The results showed that the level of p53 mRNA in esophageal epithelium of KO mice was significantly lower than that of Loxp mice, which also proved that tissue-specific Cre recombinase was expressed only in esophageal epithelium, tongue, and stomach and p53 was knocked out in the corresponding parts (Figure 2(a)).To further identify p53-knockout degree in mice esophageal mucosa, we examined the p53 protein level in mice esophageal mucosa of KO mice and Loxp mice by using the immunohistochemical staining technique. The result revealed that, compared with Loxp mice, the p53 staining of esophageal mucosa in KO mice was significantly weaker (Figure 2(b)). The positive rates of p53 protein in esophageal epithelial cells of Loxp mice and KO mice were 88.66% ± 2.93 and 47.02% ± 6.36, respectively.

We also found that p53 KO mice were more susceptible to lesions than Loxp mice. The esophageal tissues of Loxp and KO mice were taken for HE staining when the mice were 20 weeks old. Microscopically, the thickness of the esophageal mucosa of KO mice was observed, the basal cells were significantly increased and arranged disorderly, and the nucleus polarity was changed. However, there was no abnormal change in the esophageal tissue of Loxp mice: the esophageal squamous epithelial cells were arranged neatly and orderly, the epithelial layer was thin and even, the nucleus was round or elliptical, and the basal cell layer was regular (Figure 2(c)). Thus, the result showed that the p53-deficient mouse esophagus is more prone to hyperplasia, and the tumor suppressor gene *p53* plays an important role in inhibiting tumorigenesis.

### 3.3. NMBA Induces More Severe Esophageal Precancerous Lesions in KO Mice

In order to obtain an animal model of esophageal precancerous lesions within a certain period of time, we used NMBA, which is a recognized esophageal cancer inducer, to induce p53-specific knockout mice in esophageal epithelial tissues and then studied the role of p53 in the development of esophageal precancerous lesions. With reference to the diagnostic criteria for epithelial dysplasia, we divided esophageal lesions into simple grade, mild dysplasia, moderate dysplasia, severe dysplasia, carcinoma in situ, and invasive carcinoma. The HE staining sections of the esophagus were observed in all experimental mice at 16 and 48 weeks after the first injection and recorded the degree of lesions and the number of lesions (Table 1).

**Figure 3:**
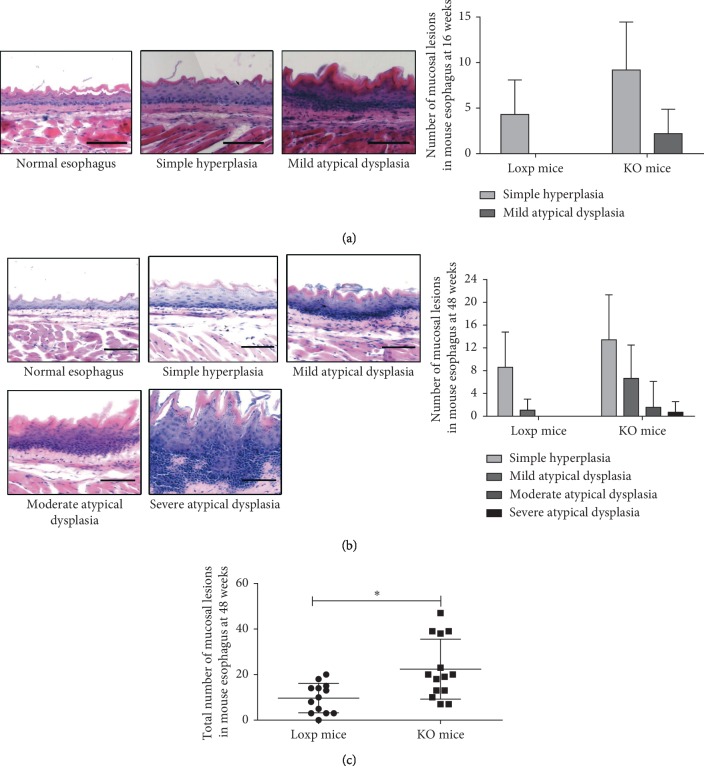
NMBA-induced esophageal precancerous lesions in KO mice. (a) Classification of mouse esophageal (HE-stained) lesions at 16 weeks and the number of the lesions grade. (b) Classification of mouse esophageal (HE-stained) lesions at 48 weeks and the number of the lesions grade. (c) Total lesions of mouse esophageal tissues at 48 weeks: the esophageal lesions of the two groups were accumulated separately (^*∗*^ means *p* < 0.05; scale bar, 100 *μ*m).

The statistical results of HE-stained sections show that NMBA-treated Loxp mice group and KO mice group showed hyperplastic lesions when the mice were fed for 16 weeks. The number of lesions in the KO mice group was significantly larger than that in the Loxp mice group (Table 1), and the KO mice group has developed mild atypical hyperplasia, while the Loxp mice group had only simple hyperplasia (Figure 3(a)). When the mice were fed for 48 weeks after the first injection, the degree of lesions in the p53 KO mice group treated with NMBA showed severe dysplasia, while the degree of lesions in the control group treated with NMBA was only mild dysplasia (Figure 3(b)) and the number of lesions at all levels in the KO mice group was significantly larger than that in the control group (Figure 3(c)). These results suggest that NMBA induces more severe esophageal precancerous lesions in KO mice.

**Figure 4:**
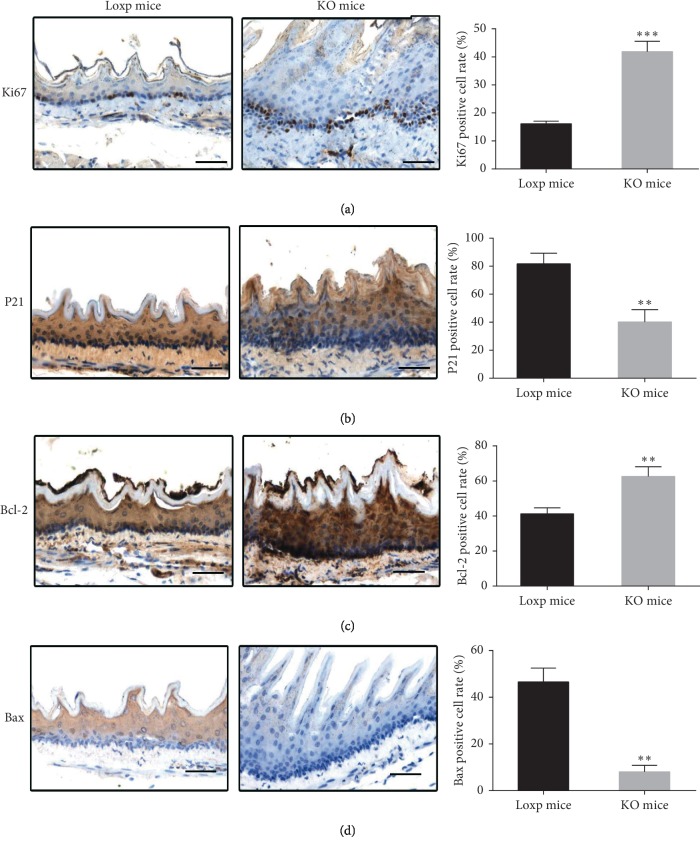
Expression of Ki67, P21, Bax, and Bcl-2 proteins in esophageal mucosal epithelial tissues of 48-week-old mice detected by IHC. (a) Ki67, (b) P21, (c) Bcl‐2, and (d) Bax (^*∗*^ means *p* < 0.05, ^*∗∗*^ means *p* < 0.01, ^*∗∗∗*^ means *p* < 0.001; scale bar, 100 *μ*m).

In addition, we also detected the expression of Ki67, P21, Bax, and Bcl-2 proteins in esophageal mucosal epithelial tissues by immunohistochemistry. Proliferation-related protein Ki67 was mainly expressed in the nucleus, cycle-regulated protein P21 was expressed in the nucleus and cytoplasm, and proapoptotic factor Bax and antiapoptotic factor Bcl-2 were mainly expressed in the cytoplasm. Immunohistochemical staining showed that the expression site (nucleus or cytoplasm) had brown substances. Compared with control group treated with NMBA, the expression of Ki67 protein and Bcl-2 protein was significantly increased (Figures 4(a) and 4(c)) and the expression of P21 protein and Bax protein was significantly reduced in the p53 KO mice group (Figures 4(b) and 4(d)).

### 3.4. Small Interfering RNA- (siRNA-) Mediated Knockdown of p53 Expression Strengthens Anchorage-Independent Growth of SHEE Cells

**Figure 5:**
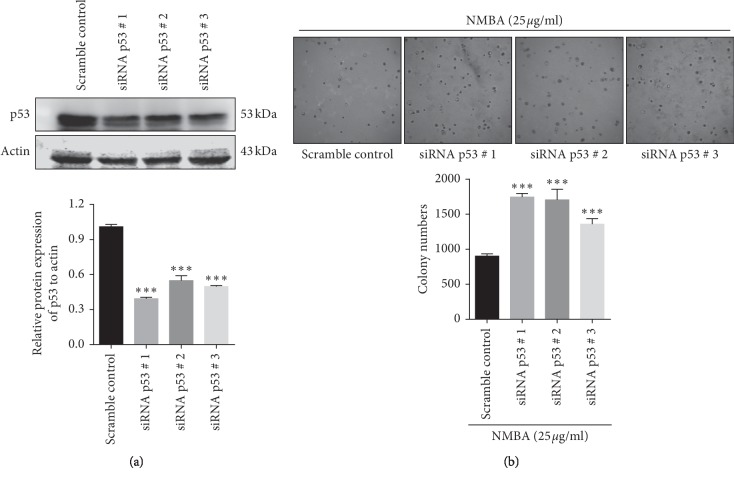
NMBA significantly enhanced the ability of p53-knockdown SHEE cells to proliferate. (a) Interference effect of siRNA on the p53 protein expression level in SHEE cells. Scramble siRNA as a negative control is a noninterfering messy oligonucleotide. (b) Soft agar colony-formation assays show that NMBA treatment increased anchorage-independent growths of p53-knockdown immortalized esophageal epithelial cells (^*∗∗*^ means *p* < 0.01, ^*∗∗∗*^ means *p* < 0.001).

In order to verify the effect of malignant transformation of p53-deficient esophageal epithelial cells under the action of NMBA in vitro, siRNA was applied to SHEE cells. After 48 h, the total protein was collected from the cells, and the interference of p53 protein was detected by Western blotting (Figure 5(a)). Scramble siRNA was a noninterfering and disordered oligonucleotide as a negative control. The constructed p53 knockdown SHEE cells were stimulated with 25 *μ*g/ml NMBA for 48 h, then plated in agar-containing medium, and cultured in a 37°C incubator for 8 d. Images were captured using a microscope (magnification, 4x).

Compared with the control group, the number and size of clones of SHEE cells of p53 knockdown induced by NMBA were significantly increased (*p* < 0.05). This result showed that NMBA can enhance the proliferation ability of p53 knockdown cells (Figure 5(b)).

## 4. Discussion

Esophageal cancer is one of the most difficult malignances to cure. However, the prognosis of esophageal cancer is favorable if it can be treated in the early stage [23]. Studying the pathogenesis of precancerous lesions in esophageal cancer will help to find chemoprevention targets instead of surgical treatment, thereby reducing the patient trauma and treatment pain. Jenkins et al. [24] found that NMBA-treated overexpressed cyclinD1 mice significantly increased esophageal hyperplasia and the level of the expression of cell proliferation antigen (PCNA) was significantly elevated. Shirai et al. [25] showed that p53 deficiency were susceptible to esophageal tumorigenesis after methyl-N-amylnitrosamine-induced p53 knockout mice. Although many studies [26, 27] are underway on esophageal cancer, the combined effects of NMBA and p53 have been rarely reported especially via p53 conditional knockout mice in esophageal epithelium. In this study, we established a mice model of esophageal precancerous lesions by specific knockout of p53 in the esophageal epithelium and induction of NMBA.

The *p53* gene was significantly knocked down in the esophageal mucosa. Meanwhile, the expression of the p53 mRNA level in the esophageal muscle layer remained almost unchanged compared with the Loxp mice group. The *p53* gene in the esophageal mucosa was specifically knocked out which showed that various types of hyperplasia and NMBA exacerbated the lesion progression. These data suggest that p53-deficient esophageal mucosa was more sensitive to the microenvironment (NMBA).

The present study has demonstrated that p53-KO mice in esophageal epithelium are more susceptible to esophageal precancerous lesions induced by NMBA than mice of genotype p53^flox/flox^Cre^−/−^. Even in the absence of NMBA, P53-deficient mice showed hyperplasia of the esophageal mucosal epithelium (Figure 2(c)). These results indicate that the deletion of *p53* gene plays an important role in the development and progression of ESCC. To further investigate the mechanisms of precancerous lesions, we examined the proteins of esophageal epithelial tissues related to the proliferation, cycle, and apoptosis by immunohistochemistry. The expression of Ki67, as a proliferation marker [28], in the esophageal mucosa of the NMBA-treated P53 knockout group increased significantly. Ki67 proteins may serve as biomarkers for early identification of esophageal cancer in the high-risk populations [29]. P21 (cyclin-dependent kinase inhibitor 1A, CDKN1) expression of esophageal tissue is weaker in the KO mice group than that in the control group, which is associated with tumor differentiation [30]. Bax and Bcl-2 proteins promote apoptosis and inhibit apoptosis, respectively [31, 32] and exhibit a negative correlation [33, 34]. In KO mice, the expression of Bcl-2 protein increased significantly. These results suggest that the mechanism of precancerous lesions of the esophagus involves the mechanisms of proliferation, cycle, and apoptosis [35]. These changes about the three proteins were consistent with clinical patient specimens [36, 37].

As a major tumor suppressor, p53 tightly controls cell proliferation, senescence, DNA repair, and cell death [38]. It is activated in response to DNA damage stress signals such as NMBA (Supplementary [Supplementary-material supplementary-material-1]). To further verify the relationship between p53 deletion and NMBA induction and esophageal cancer, we knocked down *p53* gene in normal esophageal cells. Low expression of *p53* gene obviously increased the colony-formation ability of SHEE cells. This indicated that the proliferation of p53 knockdown cells was significantly enhanced under the induction of NMBA, which is consistent with mice experiments.

In summary, our study demonstrates that the mice whose *p53* genes were specifically knocked out in the esophageal epithelium involve a multistage progression from simple hyperplasia, mild atypical dysplasia, moderate atypical dysplasia to severe atypical dysplasia under the induction of NMBA, which is similar to the pathological process of human precancerous lesions of ESCC. This precancerous lesion model can be used as a powerful tool to understand the mechanism of the early occurrence of esophageal cancer.

## 5. Conclusions

We established esophageal precancerous lesion model by using p53 conditional knockout mouse in esophageal epithelium. This precancerous lesions model is well suited for exploring the mechanism of esophageal cancer and finding a new compound for chemoprevention.

## Figures and Tables

**Table 1:** NMBA-induced statistics of lesions in esophageal epithelial carcinogenesis in mice (mean ± SD).

Time (w)	Group	Number	Simple hyperplasia	Mild atypical dysplasia	Moderate atypical dysplasia	Sever atypical dysplasia
16	Loxp mice	3	4.30 ± 3.79	0	0	0
	KO mice	5	9.20 ± 5.26	2.20 ± 2.68	0	0
48	Loxp mice	13	8.62 ± 6.17	1.07 ± 1.93	0	0
	KO mice	14	13.43 ± 7.9	6.64 ± 5.87	1.57 ± 4.54	0.71 ± 1.86

## Data Availability

The data used to support the findings of this study are available from the corresponding author upon request.

## Supplementary Materials

Supplementary Materials Loxp mice are obtained as described in the methods of KO mice model. To investigate the effect of NMBA on the p53 expression in esophageal mucosa of normal mice, the esophagus of Loxp mice injected with NMBA and sterile water, respectively, were observed by immunohistochemistry (see Supplementary Figure 1 in the Supplementary Material). The results of Supplementary Material indicate that NMBA upregulates the expression of p53 in the mucosal layer of Loxp mice. (A) There were two groups of Loxp mice that can express p53 protein. One was injected with sterile water (control) and the other was injected subcutaneously with NMBA three times a week for five weeks. The expression of p53 in the esophageal mucosal was detected by IHC. Arrows refer to the esophageal mucosa. (B) Quantification of p53 expression showed that NMBA treatment can upregulate the p53 expression (^*∗*^ means *p* < 0.05; scale bar, 100 *μ*m).
