# Supplementary material for: The Establishment of Esophageal Precancerous Lesion Model by Using p53 Conditional Knockout Mouse in Esophageal Epithelium
Source: Biomed Res Int. 2020 Jan 23;2020:4534289. doi: 10.1155/2020/4534289 (PMC7003290; doi:10.1155/2020/4534289)
Supplement: Supplementary Materials — Loxp mice are obtained as described in the methods of KO mice model. To investigate the effect of NMBA on the p53 expression in esophageal mucosa of normal mice, the esophagus of Loxp mice injected with NMBA and sterile water, respectively, were observed by immunohistochemistry (see Supplementary Figure 1 in the Supplementary Material). The results of Supplementary Material indicate that NMBA upregulates the expression of p53 in the mucosal layer of Loxp mice. (A) There were two groups of Loxp mice that can express p53 protein. One was injected with sterile water (control) and the other was injected subcutaneously with NMBA three times a week for five weeks. The expression of p53 in the esophageal mucosal was detected by IHC. Arrows refer to the esophageal mucosa. (B) Quantification of p53 expression showed that NMBA treatment can upregulate the p53 expression (∗ means p < 0.05; scale bar, 100 μm). [file 4534289.f1.pdf]

**Supplementary Material**

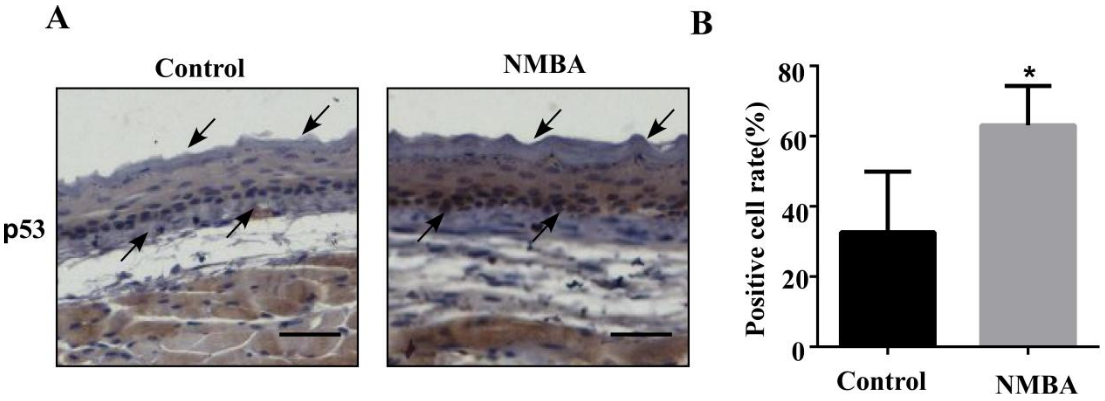


Supplementary Figure1. NMBA can up-regulate the expression of p53 in the esophageal mucosal layer of Loxp mice. (A) There were two groups of Loxp mice that can express p53 protein. One was injected with sterile water (Control) and the other was injected subcutaneously with NMBA three times a week for five weeks. The expression of p53 in the esophageal mucosal was detected by IHC. Arrows refer to the esophageal mucosa. (B) Quantification of p53 expression showed that NMBA treatment can up-regulate p53 expression. (* means $p< 0.05$; Scale bar, 100 μm).
